# Identification of differences in health impact modelling of salt reduction

**DOI:** 10.1371/journal.pone.0186760

**Published:** 2017-11-28

**Authors:** Marieke A. H. Hendriksen, Johanna M. Geleijnse, Joop M. A. van Raaij, Francesco P. Cappuccio, Linda C. Cobiac, Peter Scarborough, Wilma J. Nusselder, Abbygail Jaccard, Hendriek C. Boshuizen

**Affiliations:** 1 National Institute for Public Health and the Environment, Bilthoven, the Netherlands; 2 Division of Human Nutrition, Wageningen University, Wageningen, the Netherlands; 3 WHO Collaborating Centre, Warwick Medical School, University of Warwick, Coventry, United Kingdom; 4 University Hospitals Coventry & Warwickshire NHS Trust, Coventry, United Kingdom; 5 Centre for Health Policy, School of Population and Global Health, The University of Melbourne, Carlton Australia; 6 Centre on Population Approaches for Non-Communicable Disease Prevention, Nuffield Department of Population Health, University of Oxford, Oxford, United Kingdom; 7 Department of Public Health, Erasmus University Medical Center Rotterdam, Rotterdam, the Netherlands; 8 UK Health Forum, London, United Kingdom; State University of Rio de Janeiro, BRAZIL

## Abstract

We examined whether specific input data and assumptions explain outcome differences in otherwise comparable health impact assessment models. Seven population health models estimating the impact of salt reduction on morbidity and mortality in western populations were compared on four sets of key features, their underlying assumptions and input data. Next, assumptions and input data were varied one by one in a default approach (the DYNAMO-HIA model) to examine how it influences the estimated health impact. Major differences in outcome were related to the size and shape of the dose-response relation between salt and blood pressure and blood pressure and disease. Modifying the effect sizes in the salt to health association resulted in the largest change in health impact estimates (33% lower), whereas other changes had less influence. Differences in health impact assessment model structure and input data may affect the health impact estimate. Therefore, clearly defined assumptions and transparent reporting for different models is crucial. However, the estimated impact of salt reduction was substantial in all of the models used, emphasizing the need for public health actions.

## Introduction

The World Health Organization (WHO) recently estimated that dietary risk factors accounted for 11.3 million deaths and 241.4 million disability-adjusted life years (DALYs) [1], with high salt intake being a major contributor [2]. The WHO has set a target to reduce population salt intake by 30%, aiming at an average of 5 gram per day, by 2025 [3]. Sodium reduction in processed foods, and raising awareness of consumers on salt reduction and monitoring salt consumption in populations and food reformulations are the primary interventions to reduce the level of salt intake [4]. For several countries, the expected health gain (e.g. averted morbidity or DALYs) related to salt reduction have been calculated [5–9]. These studies used different approaches to quantify the health impact of salt reduction. Differences in modelling approaches can affect the estimated number of incident cases of disease averted or the number of deaths postponed. To illustrate, Coxson *et al* demonstrated that a 3-gram lower salt intake could avert 280,000 deaths in the USA using a dynamic-state transition model that estimated salt reduction on blood pressure and subsequently on mortality, but this number almost doubled (500,000 deaths) when a direct effect on mortality was estimated using relative risks from a post-hoc observational analysis of an randomized controlled trial of sodium reduction [8]. In otherwise comparable quantitative health impact assessment (HIA) models, these estimates may also lead to heterogeneity in estimated health outcomes. For example, Scarborough *et al* observed that applying the assumptions of the CHD policy model leads to a calculated 8 to 16% of CVD deaths being postponed, while a similar analysis using the PRIME model (previously the DIETRON model) suggested a postponement of 4 to 6% [10]. Such heterogeneities may be due to variation in underlying assumptions on the salt intake to health effect association or due to intrinsic factors of the models. Therefore, insight in the underlying model structures, assumptions and (demographic) input data used is essential to interpret and compare the outcomes of population health models.

The objective of the present study is to gain insight in how differences in various HIA models may result in heterogeneity in health impact estimates. We first identified eight models used to calculate the health impact of salt reduction, and describe their differences. In a following step we used the population health modelling tool DYNAMO-HIA to estimate to what extent the variation in the modelling assumptions and (demographic) input data used in these models affect the outcome of health impact estimates.

## Materials and methods

### Selection of models

We searched PubMed for research papers that calculated the long-term health impact of salt reduction published until August 2013, using ‘salt reduction’, ‘health impact assessment’ and ‘modelling study’ as key words. We limited our search to five models: CHD policy model [8, 11]; PRIME model [10]; Proportional Multistate Life-Table (PMLT) [7]; Global burden of disease (GBD) [2] and RIVM-CDM [12].

We also identified three additional salt reduction models that fulfilled the above mentioned criteria, but at the time of our search the results had not yet been published (IMPACT model [13], DYNAMO-HIA model [14] and UK Health Forum model. Since the UK Health Forum Model on salt reduction is not yet published in a peer-reviewed journal, our present analyses concern seven models.

### Identification of model features

**Fig 1:**
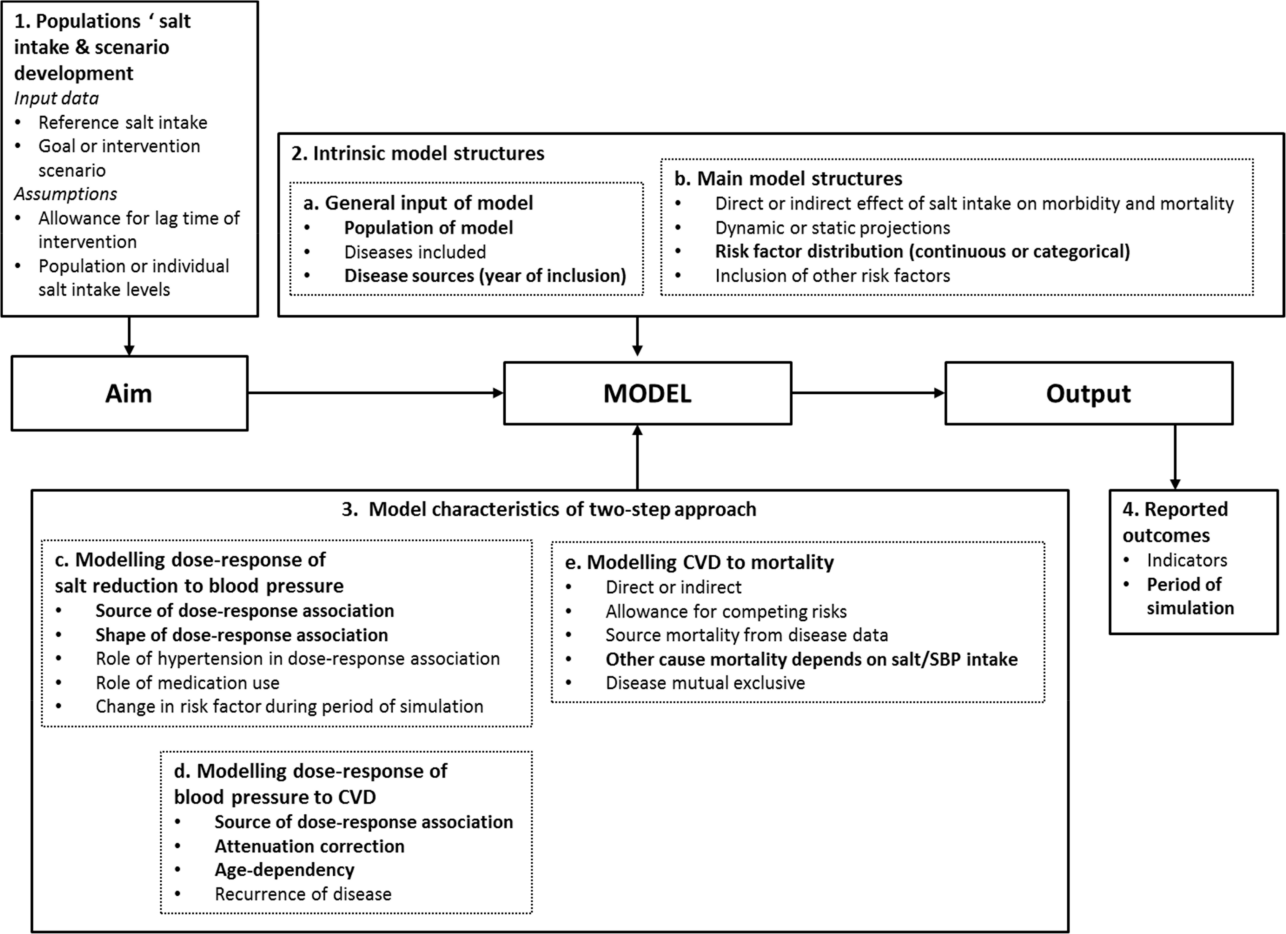
Four key features and its underlying assumptions and input data of the modelling approaches of salt reduction.

We identified four key features of the models related to the aim of the study, the characteristics of the quantitative impact assessment model used and the output obtained (Fig 1). In our view the most relevant data needed in the model and assumptions that need to be made are clustered in seven boxes in Fig 1 for each feature. In Table 1 we described for each model which (demographic) input data were used and how assumptions were worked out.

**Table 1:** Comparison of main model features of the models that calculated health impact of salt intake reduction.

Modelling approaches	CHD policy model [8, 11]	Proportional multistate life table [7]	RIVM-CDM [12]	PRIME Model [10]	IMPACT model [13]	Global burden of disease [15]	DYNAMO-HIA [14]
**Population salt intake & scenario development**							
*Goal*	1 g/d reduction; 2 g/d reduction; 3 g/d reduction	4 specific interventions	2 specific interventions and goal intake to 6 g/d	Goal: 6 g/d	2–20% intake reduction due to specific interventions	Theoretical minimum risk exposure	30% reduction; Goal: 5 g/d
*Lag times in scenario*	Gradual reduction in sensitivity analyses	No	No	No	1 year after baseline	No	No
*Salt intake levels*	Population level & population shift	Population level & population shift	Individual level and individual shift	Population level & population shift	Population level	Population level	Population level and shift
**General input data of model**							
*Population of model*	35-80y	>30y	>20y	<75y	Total population	Total population	>18y
*Diseases included*	Cardiac arrest, MI, CHD and stroke	IHD, stroke	AMI, CVA, CHF	IHD, stroke, stomach cancer	AMI, post AMI, HF, angina, post revascularisation	Stomach cancer, IHD, strokes, several other CVD, chronic kidney disease	IHD, stroke
*Disease sources (year)*	Prevalence: Survey Incidence: hospital register, MI registry (2000) from USA	Australian burden of disease (<2008 and trends to 2020)	Dutch GP and Hospital register (2007)	UK cause-specific mortality (2007)	Hospital statistics, MI audit project, GP-register from UK (1993–2010 and predicted to 2020)	DISMOD-MR(3) (2010)	Dutch GP registry (2003)
**Main model structures**							
*Effect of salt on CVD/other disease*	Indirect	Indirect	Indirect	Indirect	Indirect	Indirect (SBP-CVD) and direct (stomach cancer)	Indirect
*Projections*	Dynamic	Dynamic	Dynamic	Static	Static	Static	Dynamic
*Risk factor distribution*	Categorical	Continuous	Continuous (salt); Categorical (SBP)	Continuous	Continuous	Continuous	Categorical (salt) and continuous (SBP)
*Other risk factors*	Yes, multiplicative	Not used (but optional)	Not used (but optional)	Yes, multiplicative	No	No	No
**Modelling dose-response of salt intake on blood pressure**							
*Source of dose-response association*	He & MacGregor, 2004 [16] for low risk estimate and [17, 18] for high risk estimates	Law et al, 1991 [19]	He & MacGregor, 2004 [16]	He & MacGregor, 2008 [20]	He & MacGregor, 2004 [16]	Own meta-analysis based on He and MacGregor 2008 and Graudal et al, 2011 [21]	He & MacGregor, 2004 [16]
*Shape*	Linear	Exponential	Exponential	Linear	Linear	Linear	Exponential
*Role of hypertension*	By hypertension; >65 years is hypertension	Depends on SBP level	Depends on SBP level	In normotensives only, age-dependent from DASH trial	By hypertension	By age	Depends on SBP level
*Medication use*	Medication is treated similar as hypertension	Ignored	Ignored	Ignored	Ignored	Ignored	Ignored
*Change in risk factor during modelling period*	Unchanged	Unchanged	Unchanged	N/A	N/A	N/A	Unchanged
**Modelling dose-response of blood pressure to CVD**							
*Source of dose-response association*	Framingham risk scores [22],	Prospective Studies Collaboration [23]	Prospective Studies Collaboration and own meta-analysis (CHF) [23]	Prospective Studies Collaboration [23]	INTERHEART, [24]	Prospective Studies Collaboration [23] for CVD	Prospective Studies Collaboration [23]
*Attenuation correction*	No	Yes	Yes	No	No	No	Yes
*Age-dependent*	No (age effect not significant)	Yes	Yes	Yes	Yes	Yes	Yes
*Recurrence of disease*	Ignored	The lag option is based on WHO assumption of full reversal of stroke risk after 3 years, and two-thirds reversal of heart disease risk after 3 years, with the remaining heart disease risk reversed over seven subsequent years.	Ignored	Ignored	Ignored	Ignored	Ignored
**Modelling effect of CVD to mortality**							
*Direct or indirect*	Indirect (including direct fatality)	Indirect	Indirect	Direct	Direct	Direct	Indirect
*Competing risks*	Yes	Yes	Yes	N/A	N/A	N/A	Yes
*Source mortality from disease data*	Framingham adjusted for trends in risk factors and calibrated to national cause of death data; specific data sources separating out over categories	Australian burden of disease	Record linkage of Dutch GP registry and hospital register	N/A	Median survival, estimated 2020 mortality	DISMOD-MR	GP registry
*Mortality depends on salt intake/SBP before diseases*	Yes	No	No	N/A	N/A	N/A	No
*Mortality depends on salt intake/SBP after disease*	No	No	No	N/A	N/A	N/A	No
*Diseases mutual exclusive*	Partly	No (independent)	No (independent)	N/A	Yes	One at the time	No (independent)
**Reported outcomes**							
*Indicator*	Incidence, all-cause mortality and QALYs	DALY, lifetime mortality and morbidity	LYG, DALY, incidence and mortality	Cause-specific mortality	LYG, DPP	DALY (YLL, YLD)	Prevalence, mortality and DALYs
*Period of simulation*	10y	Lifetime	20y	N/A	10y	N/A	20y

LYG: life years gained; DPP: deaths prevented or postponed; QALY: quality adjusted life years; DALY: disability adjusted life years; YLD: years lived with disease; YLL: years lived lost

### Selection of input data or assumptions for modelling exercise

We selected the DYNAMO-HIA model to calculate the effect of the modification of the (demographic) input data and assumptions on the health impact estimates. A detailed description of the model can be found elsewhere [14, 25]. We identified those input data and assumptions within sets of key features that differed between the selected models. Subsequently, we evaluated which input data and assumptions could be modified in the DYNAMO-HIA approach. The ten selected input data and assumptions are marked bold in Fig 1. Some other assumptions or (demographic) input data differed between the selected models, but those assumptions are too closely related to the model structure or could not be modulated in another modelling setting. For example, a model may provide either dynamic or static projections. Static models have no dimension of time, while dynamic models make it possible to estimate changes over time, and as such take into account competing risks. Such a structure cannot be modified. Therefore such differences were not examined in the present analysis. We also choose not to model the impact of changing any input data or assumptions related to the characteristic “Population salt intake and scenario development”.

### Varying modifiable input data and assumptions using DYNAMO-HIA

The selected assumptions were modified so that new input parameters were obtained. The shape and the source of the dose-response association for salt reduction to blood pressure were combined into a single input parameter. We also adapted input data, such as the age of the population, disease sources and time frame of the simulation. In an additional simulation we mirrored the CHD policy model approach [11] in DYNAMO-HIA, using the following input 1) age range of the population from 35 to 80 years; 2) relative risks for the salt to SBP relation from a meta-analysis of randomized controlled trials; 3) change of mean blood pressure levels within categories; 4) relative risks for the BP to CVD relation from the Framingham Study and 5) ‘other cause mortality’ (that is, other causes than the modelled diseases IHD and stroke) depending on current blood pressure levels. We used DYNAMO-HIA version 2.07. In all situations, we simulated the health impact of a 3-gram salt reduction for the Dutch population in a closed cohort. The default situation of the DYNAMO-HIA approach and the alternative simulations are presented in Table 2. The alternative simulations were each compared with the default situation. For each simulation, we report the effect on the incidence of stroke and ischemic heart disease (IHD). We also estimated the effect on the life expectancy (LE) for a 60-year old individual. An overview of relative risks used in the calculations is presented in the supplementary information.

**Table 2:** Overview of the assumptions and input data within the DYNAMO-HIA approach (default situation) and its modifications in the alternative simulations.

Features	Default situation	Alternations compared to default situation
*General input of model*		
Population of model	> 18 years	35–80 years
Disease sources	GP registries, 2001	GP registries and hospital registration from 2010
*Main model structures*		
Risk factor distribution	Categorical for salt intake (per 2 g salt), but continuous blood pressure distribution	Categorical for salt intake (per 2 g salt), and categorical for blood pressure (per 20 mmHg)
		Changing prevalence of population in SBP categories (RIVM-CDM approach)
		Change of mean blood pressure in SBP categories (CHD policy approach)
*Modelling dose-response of salt reduction to blood pressure*		
Shape and source of dose-response association	He and MacGregor, 2004; Exponential	Law et al, 1991;Linear
*Modelling dose-response of blood pressure to CVD*		
Source of dose-response association	Prospective Studies Collaboration, 2002, age-specific	Framingham Risk Estimates, unadjusted for age
Attenuation correction	Measured blood pressure adjusted for within-subject variation	Measured blood pressure
Age-dependent	Yes	No age-dependency using Framingham risk estimates
*Modelling effect of CVD to mortality*		
Other cause^1^ of death mortality depends on salt intake/SBP	No	Yes
*Reported outcomes*		
Period of simulation	10 years	Extended to 20 years
		Extended to 50 years

^1^Other then stroke and IHD

## Results

### Model features and its underlying assumptions and input data

Table 1 shows the assumptions and input data of the main model features for all seven models. With respect to the population salt intake and scenario development, we observed that all models simulated a salt reduction scenario: estimating the effect of an intervention or a fixed target. In the scenarios, no lag time of the intervention scenario was assumed, except for the CHD policy and IMPACT model, and a change in salt intake at population level was estimated, except for RIVM-CDM where individual shifts in salt intake were used. With respect to the general input data of the model, the age range of the population exposed to the intervention also differed. All models estimated the effect of salt reduction on CVD mediated by SBP. GBD and PRIME included diseases other than CVD. Prevalence and incidence data of the diseases at the start of the simulation, necessary as input for the models, were based on country-specific registries and databases. Concerning the main model structures, we identified four dynamic (CHD policy model, RIVM-CDM, DYNAMO-HIA and PMSLT) and three static models (PRIME, IMPACT and GBD). Salt intake and/or systolic blood pressure (SBP) were divided into categories in the CHD policy model, RIVM-CDM and DYNAMO-HIA, but were considered continuous in the other models. The effect of SBP changes over categories was approached differently between RIVM-CDM and the CHD policy model. In RIVM-CDM, the prevalence of the population within the SBP categories changed. In the CHD policy model the mean SBP within each SBP was decreased within each SBP category.

Five HIA models derived the salt-blood pressure relation from the same meta-analysis of RCTs [16] and one (PMSLT) from a meta-analysis of observational studies [19]. The CHD policy approach obtained two dose-response relationships from two types of studies. The first is based on the meta-analysis of randomized controlled trials [16], and a second is based on data from clinical trials [17, 18]. The dose-response relation obtained from the similar publication of He and MacGregor [16] could be interpreted as a separate linear dose-response relations for normotensive and hypertensive subjects (CHD Policy model, GBD, PRIME and IMPACT), or could be constructed in an exponential dose-response relationship that depended on blood pressure (PMLST, RIVM-CDM and DYNAMO-HIA). The association between blood pressure and CVD was derived in most models (RIVM-CDM, PMSLT, DYNAMO-HIA, PRIME and GBD) from the Prospective Studies Collaboration [23]. RIVM-CDM, PMLST and DYNAMO-HIA adjusted the variance of the measured SBP levels of the population for the within-subject variability (attenuation correction). PRIME and GBD used only the population-average SBP levels and not its variance. The CHD policy model and the IMPACT model obtained the effect size of the SBP to CVD morbidity relationship from specific cohort studies, namely the Framingham Cohort Study and the INTERHEART Study [22] [24].

A combination of disease incidence and mortality and integrative measures (such as DALYs) are mostly reported as outcome measure. The period of simulation varies between 10 years (CHD policy model and IMPACT model) to lifetime (PMSLT).

### Effect of the modifications of input data or assumptions on estimated health impact

**Fig 2:**
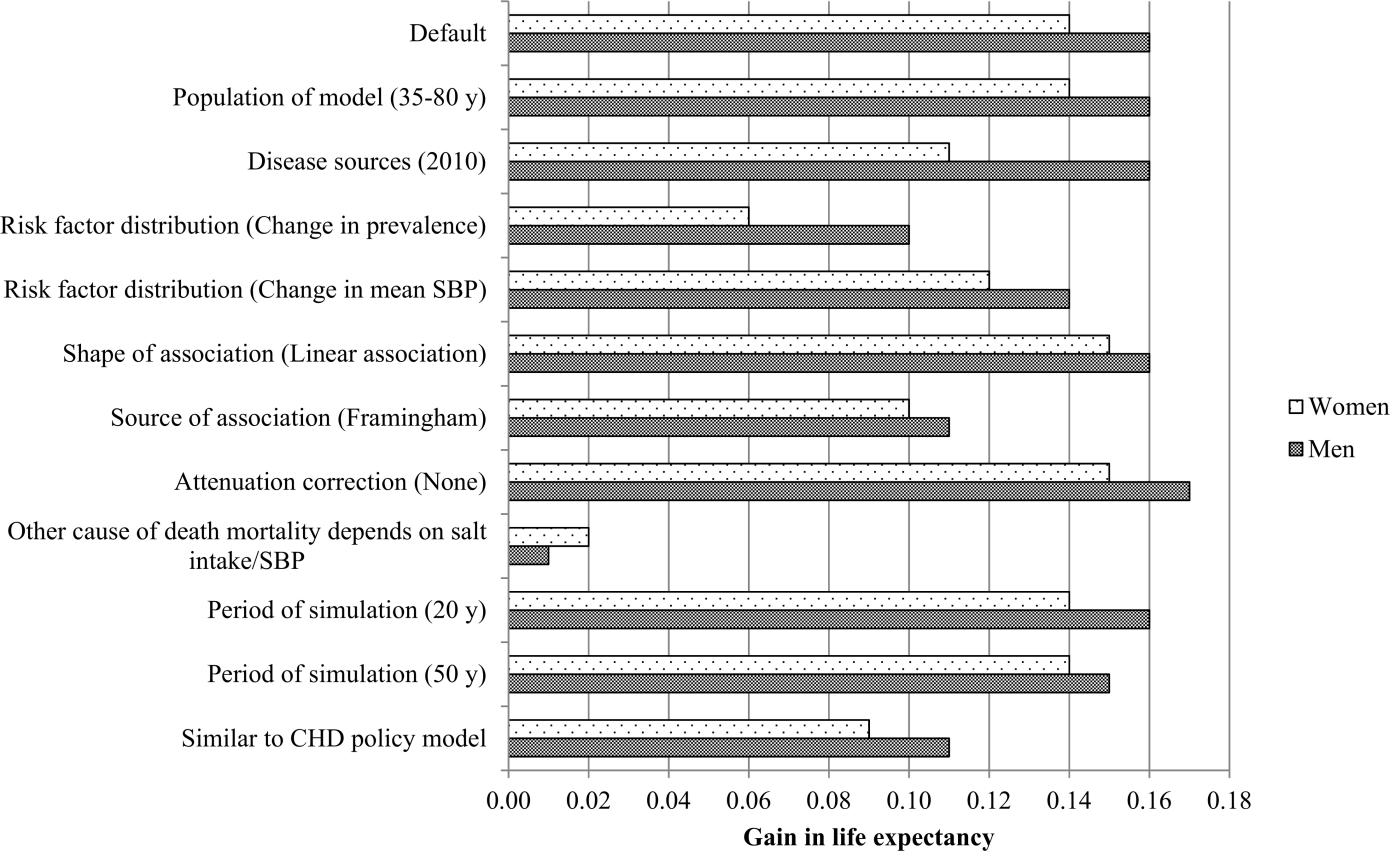
Gain in life expectancy for men and women aged 60 between 3 gram salt reduction and current salt intake for the various simulations.

Table 3 shows the various estimates of the impact of a 3-gram salt reduction (using the DYNAMO-HIA model) for the population aged 18 years and older in the Netherlands applying six alternative assumptions and four variations of input data. In the default situation, after 10 years, a 3 gram salt reduction resulted in a 10.5% reduction (N = 30,800) in stroke incidence and a 7.9% reduction in IHD incidence (N = 38,100). The gain in LE was 0.16 years for men and 0.14 years for women (Fig 2).

**Table 3:** Effect of eight modifiable assumptions and input data on the health impact estimate of a 3 gram salt reduction using the DYNAMO-HIA model.

		CVA incidence				IHD incidence			
		Baseline	3 g/d salt intake reduction	Absolute difference	% reduction (% difference with default approach)	Baseline	3 g/d salt intake reduction	Absolute difference	% reduction (% difference with default approach)
	Default^1^	292,700	261,900	30,800	10.5	483,600	445,500	38,100	7.9
**General input of the model**									
Population of model	35–80 y	253,500	225,000	28,500	11.2 (+6%)	445,400	409,800	35,600	8.0 (1%)
Disease sources	CVD data from 2010	275,200	246,300	28,900	10.5 (0%)	528,000	487,000	41,000	7.8 (-1%)
**Main model structures**									
Risk factor distribution	Change in prevalence in categories	290,800	269,100	21,700	7.2 (-31%)	482,100	456,400	25,700	5.4 (-32%)
	Change in mean SBP in categories	290,800	261,300	29,500	10.1 (-4%)	482,000	444,400	37,600	7.8 (-1%)
**Modelling effect of salt reduction in blood pressure**									
Salt intake–SBP	Linear association, with RR from Law, 1991	293,400	256,200	37,300	12.7 (+19%)	483,700	437,400	46,300	9.6 (+22%)
**Modelling effect of blood pressure on CVD**									
SBP-CVD	RR from Framingham	292,900	270,900	22,000	7.5 (-33%)	483,600	460,200	23,400	4.8 (-40%)
Attenuation correction	No correction usual SBP	292,700	258,400	34,300	11.7 (+11%)	483,600	442,600	41,000	8.5 (+8%)
**Modelling effect of CVD on mortality**									
Mortality also depends on SBP directly^2^	Other cause of death mortality depends on salt intake/SBP	292,700	261,400	31,300	10.7 (+2%)	483,600	444,600	39,000	8.1 (+3%)
**Reported outcomes**									
Period of simulation	Extended to 20 y	652,400	586,400	66,000	10.1 (-4%)	1,066,700	986,500	80,200	7.5 (-5%)
	Extended to 50 y	1,889,200	1,717,800	171,400	9.0 (-14%)	2,808,100	2,621,974	186,200	6.6 (-16%)
**Combined approach**									
	Similar to CHD policy model	252,900	233,300	16,900	7.8 (-26%)	445,200	422,900	22,300	5.0 (-37%)

^1^ default situation: 10-year period, population aged >18 years and older, correction for RDR. RR salt intake and SBP from He and MacGregor et al 2004 (exponential), RR SBP-CVD Lewington et al, 2002, measured SBP corrected with regression dilution ratio

^2^The pathway from SBP to mortality in this model is both through the “indirect” effect of SBP increasing stroke and IHD incidence, and through a direct effect on mortality from other causes

The largest changes in health impact estimates were observed when risk estimates in the dose-responses association were changed. In the simulation using risk estimates for the relation between blood pressure and diseases from the Framingham Cohort Study, the absolute numbers as well as the percentage reduction of the estimated disease incidence was considerably lower compared with the default situation (7.5% (N = 22,000) reduction for stroke and 4.8% (N = 23,400) for IHD; Table 3). This means a 33% lower estimate for stroke and a 40% lower estimate for IHD compared with the default situation. The health impact estimates were higher if the linear association between salt intake and blood pressure taken from the study of Law were incorporated in the model (lower estimate of 12.5% (N = 36,500) for stroke and 9.6% (N = 46,300) for IHD). This means a 19% higher estimate for stroke and a 22% higher estimate for IHD compared to the default situation.

Other substantial differences with the default simulation occurred when the prevalence of the population in each SBP categories shifted as a consequence of salt reduction (31% lower estimate for stroke and 32% lower estimate for IHD as compared to the default situation; see Table 3). Other changes have a small impact, such as no correction for usual blood pressure. Extending the calculations to 20 or to 59 years does not have an effect on percentage change, but the absolute number of incident cases is much higher when the calculations are extended to 20 or to 50 years. The combined approach with modifications similar to the CHD policy model led to a stroke reduction of 7.8% and to an IHD reduction of 5.0%. This means a 26% lower estimate for stroke and a 37% decreased estimate for IHD compared to the default situation.

Gain in life expectancy (Fig 2) followed a similar trend as the results in Table 3.

## Discussion

Our overview of selected HIA models of salt reduction showed that despite the many differences between the models, there are important similarities. All studies examined the effect of salt intake on CVD as mediated by SBP, with substantial projected health gains (7.2% to 12.7% for stroke and 4.8% to 9.6% for IHD). Differences in assumptions between HIA models mainly concerned the strength of the relationships between salt intake and SBP, and between SBP and disease occurrence. In addition, we observed that an association obtained from literature could be interpreted differently in the modelling exercise. The models also differed in intrinsic model structures, such as categorization of salt intake and/or blood pressure levels and dynamic versus static approach.

In this study, we assessed to what extent model input data and assumptions may determine health impact estimates using a standard dynamic model (DYNAMO-HIA). In the default scenario, a 3 gram salt reduction reduced the incidence of stroke by 10.5% and the incidence of IHD by 7.9%, in the Dutch population over a period of 10 years. Changing the assumptions relating to the association between salt intake and blood pressure and between blood pressure and CVD changed the health impact estimates substantially. Changing the relative risks of blood pressure on CVD reduced the incidence by 33% for stroke and 40% for IHD. After this, using blood pressure in categories, and allowing salt intake change the proportion of the population in each category (reduction of HIA estimate by 27% for stroke and by 18% for IHD) appeared to have the most effect on the incident cases. Effects of changes in the input data had less effect; however, extending the time frame of the calculations had a large impact on the absolute number of incident cases.

This is the first study that systematically compared various indirect and complex health modelling approaches for salt reduction based on four sets of key model features and their underlying assumptions and input data. Some limitations of the study need to be addressed. First, due to the selection of predefined key elements other potential differences, such as distinctions in subgroups, have not been taken into account. Second, we selected the DYNAMO-HIA model to quantify only for the Dutch population. The estimated differences in the alternative simulations may vary if this exercise is replicated in other models or in other populations. Finally, we only varied a limited set of input data and assumptions in DYNAMO-HIA model, and thus we cannot quantify the impact of remaining differences, such as the allowance of competing risks or using a static modelling approach. The potential difference between dynamic and static models was not assessed, as it was considered an un-adjustable, intrinsic aspect of the HIA models. However, we do assume that dynamic models are more adequate to estimate future health gain as they take into account selective mortality, ageing and competing risks.

In the present study, the health impact estimates changed when input data and assumptions were replaced by alternative input data or assumptions. Three assumptions or alternative input data are the most influential on the relative and absolute outcome of the health impact assessment: the sources of relative risks used in the blood pressure to health association, the dose-response between salt intake and blood pressure and the distribution of risk factors. Because of the importance of the effect of the relative risk on the outcome, it is important that the source is obtained from good quality, prospective studies. This study showed that using categorical risk factor distributions seemed to reduce the sensitivity of the model to changes in salt intake. This is probably due to the fact that this modelling approach lowers blood pressure in all subjects lowering salt intake will decrease blood pressure in all subjects, but only a few subjects will shift to a lower blood pressure category and thereby will have a lower risk of developing CVD.

In general, uncertainty analyses show how the health impact estimate depends on the underlying assumptions and (demographic) input data within a single HIA model and is therefore helpful to identify the range of the expected effect. However, uncertainty analyses are often only applied to a limited set of model assumptions or (demographic) input data, for example the relative risks (parametric uncertainty). The present study showed that also intrinsic model structures and (demographic) input data contribute to the variation in the health impact estimates (structural uncertainty), but is rarely assessed in modelling studies. Therefore, there is a clear need for transparency in HIA models, if necessary in a technical appendix, where transparency refers to the clear description of the model structures and (demographic) input data used, and also to describe the full range of uncertainty assessed by models. Our analysis showed that it is important to develop standard reporting guidelines for the field of non-communicable disease scenario modelling.

Comparing the variation in outcomes from substituting input data or assumptions in a model one by one was informative to identify the main assumptions that could contribute to the heterogeneity in the outcomes of published studies. In practice, HIA models vary in several underlying assumptions. Mirroring our approach to the approach used by the CHD policy model resulted in an impact estimate higher than the estimate of using the CHD policy model itself (for example, stroke 7.8% for DYNAMO-HIA vs 5.2% of CHD policy model). Thus, by making approaches comparable we could not fully explain the variation between the models. Obviously, there will be some remaining differences, such as the difference in demographic and socio-economic data (such as country-specific incidence and prevalence of CVD). Therefore, a comparative study of the various models using similar input data (demographic as well as intervention scenario) could help to understand how the impact assessment differs between the various models, taking into account the mutual differences between the models.

One aspect of interest since the completion of this analysis is the growing evidence of social inequalities in salt consumption as the more disadvantaged social groups not only have the highest burden of cardiovascular disease, but also the highest salt consumption [26–29]. A reduction in salt intake would therefore be likely to exert a greater health impact in those groups. Also, newer studies have been published that estimated the health impact of salt reduction (e.g. [30–33], using similar approaches. Therefore, we do not think that inclusion of these papers will change our conclusions.

In conclusion, our study shows that especially differences in the strength and shape of the dose-response association from salt to health contributed to heterogeneity of the health impact estimates reported. We concluded that transparency of the models structures and (demographic) data used is essential to be able to interpret the outcomes of a health impact assessment. We advise to develop standard reporting guidelines for the field of non-communicable disease scenario modelling.

## Supporting information

S1 Data Supplement: Input files for DYNAMO-HIA–default analyses. (ZIP)

S2 Data Supplement: Input files for DYNAMO-HIA–change in prevalence. (ZIP)

S3 Data Supplement: Input files for DYNAMO-HIA–Law1991. (ZIP)

S4 Data Supplement: Input files for DYNAMO-HIA—New CVD data. (ZIP)

S5 Data Supplement: Input files for DYNAMI-HIA–No correction usual SBP. (ZIP)

S6 Data Supplement: Input files for DYNAMO-HIA–RR Framingham. (ZIP)

S7 Data Supplement: Input files for DYNAMO-HIA–Similar to CHD policy model. (ZIP)

S8 Data Supplement: Distribution of salt intake and blood pressure levels in the Netherlands. (ZIP)

S9 Data Supplement: R-scripts to calculate the relative risks used in DYNAMO-HIA. (ZIP)

## References

[1] Forouzanfar MH, Alexander L, Anderson HR, Bachman VF, Biryukov S, Brauer M, et al Global, regional, and national comparative risk assessment of 79 behavioural, environmental and occupational, and metabolic risks or clusters of risks in 188 countries, 1990–2013: a systematic analysis for the Global Burden of Disease Study 2013. Lancet. 2015;386(10010):2287–323. doi: 10.1016/S0140-6736(15)00128-2 26364544 10.1016/S0140-6736(15)00128-2 PMC4685753

[2] Lim SS, Vos T, Flaxman AD, Danaei G, Shibuya K, Adair-Rohani H, et al A comparative risk assessment of burden of disease and injury attributable to 67 risk factors and risk factor clusters in 21 regions, 1990–2010: a systematic analysis for the Global Burden of Disease Study 2010. Lancet. 2013;380(9859):2224–60. 10.1016/S0140-6736(12)61766-8 PMC4156511 23245609

[3] Beaglehole R, Bonita R, Horton R, Adams C, Alleyne G, Asaria P, et al Priority actions for the non-communicable disease crisis. Lancet. 2011;377(9775):1438–47 doi: 10.1016/S0140-6736(11)60393-0 21474174 10.1016/S0140-6736(11)60393-0

[4] Cappuccio FP, Capewell S, Lincoln P, McPherson K. Policy options to reduce population salt intake. BMJ. 2011;343:d4995 doi: 10.1136/bmj.d4995 21835876 10.1136/bmj.d4995

[5] Asaria P, Chisholm D, Mathers C, Ezzati M, Beaglehole R. Chronic disease prevention: health effects and financial costs of strategies to reduce salt intake and control tobacco use. Lancet. 2007;370(9604):2044–53. doi: 10.1016/S0140-6736(07)61698-5 18063027 10.1016/S0140-6736(07)61698-5

[6] Barton P, Andronis L, Briggs A, McPherson K, Capewell S. Effectiveness and cost effectiveness of cardiovascular disease prevention in whole populations: modelling study. BMJ. 2011;343:d4044 doi: 10.1136/bmj.d4044 21798967 10.1136/bmj.d4044 PMC3145836

[7] Cobiac LJ, Vos T, Veerman JL. Cost-effectiveness of interventions to reduce dietary salt intake. Heart. 2010;96(23):1920–5. doi: 10.1136/hrt.2010.199240 21041840 10.1136/hrt.2010.199240

[8] Coxson PG, Cook NR, Joffres M, Hong Y, Orenstein D, Schmidt SM, et al Mortality Benefits From US Population-wide Reduction in Sodium Consumption: Projections From 3 Modeling Approaches. Hypertension. 2013;61(3):564–70. doi: 10.1161/HYPERTENSIONAHA.111.201293 23399718 10.1161/HYPERTENSIONAHA.111.201293

[9] Gardener H, Rundek T, Wright CB, Elkind MS, Sacco RL. Dietary sodium and risk of stroke in the Northern Manhattan study. Stroke. 2012;43(5):1200–5. doi: 10.1161/STROKEAHA.111.641043 22499576 10.1161/STROKEAHA.111.641043 PMC3347890

[10] Scarborough P, Nnoaham KE, Clarke D, Capewell S, Rayner M. Modelling the impact of a healthy diet on cardiovascular disease and cancer mortality. J Epidemiol Community Health. 2012;66(5):420–6. doi: 10.1136/jech.2010.114520 21172796 10.1136/jech.2010.114520

[11] Bibbins-Domingo K, Chertow GM, Coxson PG, Moran A, Lightwood JM, Pletcher MJ, et al Projected effect of dietary salt reductions on future cardiovascular disease. N Engl J Med. 2010;362(7):590–9. doi: 10.1056/NEJMoa0907355 20089957 10.1056/NEJMoa0907355 PMC3066566

[12] Hendriksen MA, Hoogenveen RT, Hoekstra J, Geleijnse JM, Boshuizen HC, van Raaij JM. Potential effect of salt reduction in processed foods on health. Am J Clin Nutr. 2014;99(3):446–53. doi: 10.3945/ajcn.113.062018 24335058 10.3945/ajcn.113.062018

[13] Collins M, Mason H, O'Flaherty M, Guzman-Castillo M, Critchley J, Capewell S. An economic evaluation of salt reduction policies to reduce coronary heart disease in England: a policy modeling study. Value in health: the journal of the International Society for Pharmacoeconomics and Outcomes Research. 2014;17(5):517–24. 25128044 10.1016/j.jval.2014.03.1722

[14] Hendriksen MA, van Raaij JM, Geleijnse JM, Breda J, Boshuizen HC. Health gain by salt reduction in europe: a modelling study. PLoS One. 2015;10(3):e0118873 doi: 10.1371/journal.pone.0118873 25826317 10.1371/journal.pone.0118873 PMC4380413

[15] Mozaffarian D, Fahimi S, Singh GM, Micha R, Khatibzadeh S, Engell RE, et al Global sodium consumption and death from cardiovascular causes. N Engl J Med. 2014;371(7):624–34. doi: 10.1056/NEJMoa1304127 25119608 10.1056/NEJMoa1304127

[16] He FJ, MacGregor GA. Effect of longer-term modest salt reduction on blood pressure. Cochrane Database Syst Rev. 2004(3):CD004937 doi: 10.1002/14651858.CD004937 15266549 10.1002/14651858.CD004937

[17] Sacks FM, Svetkey LP, Vollmer WM, Appel LJ, Bray GA, Harsha D, et al Effects on blood pressure of reduced dietary sodium and the Dietary Approaches to Stop Hypertension (DASH) diet. DASH-Sodium Collaborative Research Group. N Engl J Med. 2001;344(1):3–10. doi: 10.1056/NEJM200101043440101 11136953 10.1056/NEJM200101043440101

[18] MacGregor GA, Markandu ND, Sagnella GA, Singer DR, Cappuccio FP. Double-blind study of three sodium intakes and long-term effects of sodium restriction in essential hypertension. Lancet. 1989;2(8674):1244–7. 2573761 10.1016/s0140-6736(89)91852-7

[19] Law MR, Frost CD, Wald NJ. By how much does dietary salt reduction lower blood pressure? I—Analysis of observational data among populations. BMJ. 1991;302(6780):811–5. 2025703 10.1136/bmj.302.6780.811 PMC1669164

[20] He FJ, MacGregor GA. Salt intake and cardiovascular disease. Nephrol Dial Transplant. 2008;23(11):3382–4; discussion 5. 18938265 10.1093/ndt/gfn550

[21] Graudal NA, Hubeck-Graudal T, Jurgens G. Effects of low sodium diet versus high sodium diet on blood pressure, renin, aldosterone, catecholamines, cholesterol, and triglyceride. Cochrane Database Syst Rev. 2011(11):CD004022 doi: 10.1002/14651858.CD004022.pub3 22071811 10.1002/14651858.CD004022.pub3

[22] D'Agostino RB Sr., Grundy S, Sullivan LM, Wilson P. Validation of the Framingham coronary heart disease prediction scores: results of a multiple ethnic groups investigation. Jama. 2001;286(2):180–7. 11448281 10.1001/jama.286.2.180

[23] Lewington S, Clarke R, Qizilbash N, Peto R, Collins R. Age-specific relevance of usual blood pressure to vascular mortality: a meta-analysis of individual data for one million adults in 61 prospective studies. Lancet. 2002;360(9349):1903–13. 12493255 10.1016/s0140-6736(02)11911-8

[24] Yusuf S, Hawken S, Ounpuu S, Dans T, Avezum A, Lanas F, et al Effect of potentially modifiable risk factors associated with myocardial infarction in 52 countries (the INTERHEART study): case-control study. Lancet. 2004;364(9438):937–52. doi: 10.1016/S0140-6736(04)17018-9 15364185 10.1016/S0140-6736(04)17018-9

[25] Boshuizen HC, Lhachimi SK, van Baal PH, Hoogenveen RT, Smit HA, Mackenbach JP, et al The DYNAMO-HIA model: an efficient implementation of a risk factor/chronic disease Markov model for use in Health Impact Assessment (HIA). Demography. 2012;49(4):1259–83. doi: 10.1007/s13524-012-0122-z 23055232 10.1007/s13524-012-0122-z

[26] Cappuccio FP, Ji C, Donfrancesco C, Palmieri L, Ippolito R, Vanuzzo D, et al Geographic and socioeconomic variation of sodium and potassium intake in Italy: results from the MINISAL-GIRCSI programme. BMJ open. 2015;5(9):e007467 doi: 10.1136/bmjopen-2014-007467 26359282 10.1136/bmjopen-2014-007467 PMC4577927

[27] Rodriguez-Fernandez R, Siopa M, Simpson SJ, Amiya RM, Breda J, Cappuccio FP. Current salt reduction policies across gradients of inequality-adjusted human development in the WHO European region: minding the gaps. Public Health Nutr. 2014;17(8):1894–904. doi: 10.1017/S136898001300195X 23924617 10.1017/S136898001300195X PMC10282349

[28] Ji C, Cappuccio FP. Socioeconomic inequality in salt intake in Britain 10 years after a national salt reduction programme. BMJ open. 2014;4(8):e005683 doi: 10.1136/bmjopen-2014-005683 25161292 10.1136/bmjopen-2014-005683 PMC4156795

[29] Ji C, Kandala NB, Cappuccio FP. Spatial variation of salt intake in Britain and association with socioeconomic status. BMJ open. 2013;3(1). 10.1136/bmjopen-2012-002246 PMC3549259 23295624

[30] Nghiem N, Blakely T, Cobiac LJ, Cleghorn CL, Wilson N., The health gains and cost savings of dietary salt reduction interventions, with equity and age distributional aspects. BMC Public Health, 2016 16: p. 423 doi: 10.1186/s12889-016-3102-1 27216490 10.1186/s12889-016-3102-1 PMC4877955

[31] Nghiem N, Blakely T, Cobiac LJ, Pearson AL, Wilson N, Health and economic impacts of eight different dietary salt reduction interventions. PLoS One, 2015 10(4): p. e0123915 doi: 10.1371/journal.pone.0123915 25910259 10.1371/journal.pone.0123915 PMC4409110

[32] Webb M, Fahimi S, Singh GM, Khatibzadeh S, Micha R, Powles J et al, Cost effectiveness of a government supported policy strategy to decrease sodium intake: global analysis across 183 nations. BMJ, 2017 356: p. i6699 doi: 10.1136/bmj.i6699 28073749 10.1136/bmj.i6699 PMC5225236

[33] Wilson N, Nghiem N, Eyles H, Mhurchu CN, Shields E, Cobiac LJ et al, Modeling health gains and cost savings for ten dietary salt reduction targets. Nutr J, 2016 15: p. 44 doi: 10.1186/s12937-016-0161-1 27118548 10.1186/s12937-016-0161-1 PMC4847342

